# Epigenetic, Metabolic, and Immune Crosstalk in Germinal-Center-Derived B-Cell Lymphomas: Unveiling New Vulnerabilities for Rational Combination Therapies

**DOI:** 10.3389/fcell.2021.805195

**Published:** 2022-01-07

**Authors:** Inna Serganova, Sanjukta Chakraborty, Samuel Yamshon, Yusuke Isshiki, Ryan Bucktrout, Ari Melnick, Wendy Béguelin, Roberta Zappasodi

**Affiliations:** ^1^ Division of Hematology and Medical Oncology, Department of Medicine, Weill Cornell Medical College, New York, NY, United States; ^2^ Human Oncology and Pathogenesis Program, Memorial Sloan Kettering Cancer Center, New York, NY, United States; ^3^ Immunology and Microbial Pathogenesis Program, Weill Cornell Graduate School of Medical Sciences, New York, NY, United States; ^4^ Parker Institute for Cancer Immunotherapy, San Francisco, CA, United States

**Keywords:** GCB-DLBCLs, epigenetics, metabolic intermediates, immune microenvironment, combination therapies

## Abstract

B-cell non-Hodgkin lymphomas (B-NHLs) are highly heterogenous by genetic, phenotypic, and clinical appearance. Next-generation sequencing technologies and multi-dimensional data analyses have further refined the way these diseases can be more precisely classified by specific genomic, epigenomic, and transcriptomic characteristics. The molecular and genetic heterogeneity of B-NHLs may contribute to the poor outcome of some of these diseases, suggesting that more personalized precision-medicine approaches are needed for improved therapeutic efficacy. The germinal center (GC) B-cell like diffuse large B-cell lymphomas (GCB-DLBCLs) and follicular lymphomas (FLs) share specific epigenetic programs. These diseases often remain difficult to treat and surprisingly do not respond advanced immunotherapies, despite arising in secondary lymphoid organs at sites of antigen recognition. Epigenetic dysregulation is a hallmark of GCB-DLBCLs and FLs, with gain-of-function (GOF) mutations in the histone methyltransferase *EZH2*, loss-of-function (LOF) mutations in histone acetyl transferases *CREBBP* and *EP300*, and the histone methyltransferase *KMT2D* representing the most prevalent genetic lesions driving these diseases. These mutations have the common effect to disrupt the interactions between lymphoma cells and the immune microenvironment, via decreased antigen presentation and responsiveness to IFN-γ and CD40 signaling pathways. This indicates that immune evasion is a key step in GC B-cell lymphomagenesis. EZH2 inhibitors are now approved for the treatment of FL and selective HDAC3 inhibitors counteracting the effects of *CREBBP* LOF mutations are under development. These treatments can help restore the immune control of GCB lymphomas, and may represent optimal candidate agents for more effective combination with immunotherapies. Here, we review recent progress in understanding the impact of mutant chromatin modifiers on immune evasion in GCB lymphomas. We provide new insights on how the epigenetic program of these diseases may be regulated at the level of metabolism, discussing the role of metabolic intermediates as cofactors of epigenetic enzymes. In addition, lymphoma metabolic adaptation can negatively influence the immune microenvironment, further contributing to the development of immune cold tumors, poorly infiltrated by effector immune cells. Based on these findings, we discuss relevant candidate epigenetic/metabolic/immune targets for rational combination therapies to investigate as more effective precision-medicine approaches for GCB lymphomas.

## Introduction

Despite the clinical success of immune checkpoint blockade (ICB) therapy in solid tumors (Zappasodi et al., 2018a; Zappasodi et al., 2018b; Ribas and Wolchok, 2018), B-cell lymphomas remain largely refractory to these treatments, with the exception of Hodgkin’s lymphoma, where PD-L1 (programmed death-ligand 1) constitutes a direct tumor target (Zappasodi et al., 2015; Ansell, 2019; Armand et al., 2021a). Moreover, while the majority of patients with relapsed/refractory B-cell non-Hodgkin lymphomas (B-NHLs) respond to CAR (chimeric antigen receptor) T-cell therapy, the duration of these responses is limited in many cases (Schuster et al., 2018; Hirayama et al., 2019). Hence, there is a need to identify and overcome the barriers that prevent successful immunotherapy in B-NHL patients*.* Although B-NHLs are diseases of the immune system and pose a completely different immunologic scenario compared to solid tumors, these malignancies arise at sites of antigen recognition where immunotherapies that precisely disengage T-cell effector functions are expected to work (Zappasodi et al., 2015; Zappasodi et al., 2018a). In fact, in the rare cases of B-NHLs where immunotherapy successfully elicits protective anti-tumor immune responses, tumor remissions can be long-lasting (Lesokhin et al., 2016; Fuca et al., 2021). Overall, this underscores the potential of immunotherapy to treat lymphomas and, at the same time, our poor understanding of the mechanisms that limit or prevent its efficacy in these diseases.

Recent efforts to improve the genetic classification of diffuse large B-cell lymphomas (DLBCLs)—the most common lymphoid malignancy in adults (Swerdlow et al., 2016)—have revealed the effect of specific driver epigenetic mutations to alter expression of T-cell immune co-receptors and/or downstream signaling molecules (Chapuy et al., 2018; Wright et al., 2020). These genetic features occur more frequently in germinal center (GC) subtypes of DLBCL and are also shared by follicular lymphoma (FL)—the second most frequent form of B-NHLs, which is also of GC origin (Morin et al., 2010; Carbone et al., 2019) (abbreviated thereafter as GCB lymphomas). Specifically, gain-of-function (GOF) mutations in the histone methyltransferase EZH2 (enhancer of zeste homolog 2) or loss-of-function (LOF) mutations in the histone acetyl transferase CREBBP (cAMP-response element binding protein (CREB) binding protein) or EP300 (E1A binding protein P300) or histone methyltransferase KMT2D (lysine methyltransferase 2D), which occur in 30–40% of these diseases, contribute to the repression of antigen presentation, IFN-γ response genes, or CD40 signaling in lymphoma cells (Mlynarczyk et al., 2019). These results suggest that escape from T-cell recognition and killing is inherent part of the GCB lymphoma oncogenic program and may be controlled at an epigenetic level in these diseases.

Epigenetic and metabolic reprogramming are usually deeply linked in cancer cells. The influence of tumor-intrinsic oncogenic signaling and tumor microenvironmental factors on the availability of metabolites that are substrates or inhibitors of epigenetic enzymes is well described (Kinnaird et al., 2016; Izzo et al., 2021). In addition, altered expression or activity of chromatin-modifying enzymes can impact directly and indirectly on cellular metabolism.

Here, we review the bidirectional relationship between epigenetics and metabolism in GCB lymphomas and its impact on the immune microenvironment. First, we focus on genetic and epigenetic characteristics of GCB DLBCLs and FLs, highlighting the most common alterations in *EZH2, CREBBP,* and/or *KMT2D* epigenetic modifiers and their function in histone modifications and chromatin remodeling. We then discuss principles linking the activity of chromatin-modifying enzymes and lymphoma metabolism and the impact of these mechanisms in anti-lymphoma immunity and disease progression. Lastly, we discuss current therapeutic interventions that could be harnessed in combination to target this metabolic-epigenetic crosstalk and potentially improve the response of GCB lymphomas to immunotherapy.

## Molecular Features Supporting GC B-Cell Development and Lymphomagenesis

During their lifetime, B cells undergo a stepwise process including activation, proliferation, differentiation, and antibody secretion, which is controlled by a specific network of intracellular signaling pathways and transcription factors (TFs) deeply regulated at epigenetic level and in response to microenvironmental stimuli. GCs comprise two histologically distinct regions: the dark zone (DZ), with very proliferative GC B cells in which immunoglobulin (Ig) class-switch recombination (CSR) and somatic hypermutation (SHM) occur, and the light zone (LZ), where non-dividing GC B cells with appropriate B cell receptors (BCRs) interact with follicular dendritic cells (FDCs) and follicular helper CD4^+^ T cells (T_FH_) cells to receive proper help for further cycling into the DZ or maturation into plasma cells (PCs) (Mesin et al., 2016). Normal GC B-cell development depends on the cooperation of epigenetic and non-coding elements to control expression of multiple genes. Recent genome-wide studies allowed to map changes in the chromatin landscape, DNA methylome, 3-dimensional interactome, and coding and non-coding transcriptomes of normal and malignant B cells (Mlynarczyk et al., 2019). DNA methylation changes more frequently occur at gene body and remote upstream regions than promoter regions, although demethylation of key B-cell TF binding sites correlates with expression of those TFs and their transcriptional programs (Andrews and Payton, 2019). In GC B cells and GCB lymphomas, activation-induced cytosine deaminase (AID), which drives somatic hypermutation, also mediates DNA hypomethylation and increased methylome heterogeneity in regions associated with essential B-cell lineage genes (Dominguez et al., 2015; Teater et al., 2018). Recent data revealed that activating and repressive histone marks, chromatin accessibility, and gene expression determine defined regulatory landscape transitions in normal development of human or murine naïve B cells toward PCs (Kania et al., 2017; Kania et al., 2019). Perturbation of this program can lead to lymphomagenesis. As an example relevant for GCB lymphomagenesis, conditional deletion of the histone acetyltransferase *CREBBP* perturbs B-cell development and accelerates the development of lymphoma in BCL2-and MYC-driven mouse models (García-Ramírez et al., 2017; Hashwah et al., 2017; Jiang et al., 2017; Zhang et al., 2017). This is largely because of the role of CREBBP in the GC reaction to counteract the repressive effects of BCL6 by H3K27 acetylation at enhancers of BCL6 target genes, thus leading to GC exit. *EZH2* histone methyltransferase—another commonly mutated epigenetic modifier in GCB lymphomas—is required for the formation and maintenance of GC reaction (Béguelin et al., 2013; Béguelin et al., 2016). Thus, several chromatin modifiers regulate key B-cell TFs to temporally regulate developmental transcriptional programs, and, when mutated, are lymphomagenic.

By genetic profiling of patient-derived DLBCLs, epigenetic regulator genes (*EZH2*, *CREBBP,* and/or *KMT2D*) were shown to help classify a subset of DLBCLs into a specific “cluster 3” (C3) (Chapuy et al., 2018) or “EZB” (*EZH2* mutation and *BCL2* translocation) (Wright et al., 2020) disease subtype. *KMT2D* is the most frequently mutated epigenetic regulator gene in DLBCL, with its mutation occurring in 24 and 28% of all DLBCL or GCB-DLBCL cases, respectively (Pasqualucci et al., 2011a; Reddy et al., 2017). Mutations in *EZH2* and *CREBBP* are also enriched in GCB-DLBCL (12 and 16%) compared with all DLBCL cases (6 vs. 11%, respectively) (Pasqualucci et al., 2011a; Reddy et al., 2017). These genetic subclassifications have critically improved our ability to stratify patients with different prognosis after standard therapy with rituximab (anti-CD20) + CHOP (cyclophosphamide, doxorubicin hydrochloride, vincristine sulfate, and prednisone) chemotherapy (R-CHOP). According to transcriptional profiling, DLBCLs were classified into cell of origin (COO) categories, where GCB-DLBCLs were found to generally associate with a more favorable outcome compared with activated-B-cell (ABC)-DLBCLs. However, C3/EZB GCB-DLBCLs show significantly worse prognosis compared to other GCB-DLBCLs and progression-free survival (PFS) as short as the worst prognostic subtypes of ABC-DLBCL (Chapuy et al., 2018). The survival disadvantage of EZB/C3 DLBCLs may be at least partially explained by the fact that the mutational characteristics of double hit (DHIT) lymphomas, an aggressive subtype of DLBCL characterized by *MYC* and *BCL2* translocation, are enriched in this category (Ennishi et al., 2019a; Sha et al., 2019; Wright et al., 2020). Indeed, the inferior survival of EZB DLBCLs was only observed in patients with EZB DLBCL expressing DHIT genetic signatures (Wright et al., 2020). MYC overexpression also affects lymphoma immunophenotype, transcriptional characteristics and metabolic conditions. C3/EZB DLBCLs without *MYC* alterations (EZB-MYC^−^) generally show LZ GCB cell-like gene expression profiles, in contrast to C3/EZB DLBCLs with *MYC* rearrangements (EZB-MYC^+^), which are enriched in DZ signatures, very likely because MYC expression promotes DZ re-entry and proliferation (Béguelin et al., 2020; Wright et al., 2020). Notably, the proliferative phenotype of EZB-MYC^+^ DLBCLs is coupled to highly glycolytic metabolism and sustained protein and lipid synthesis in contrast to EZB-MYC^-^ and other types of DLBCL (Wright et al., 2020).

The mutational landscape of FL is close to that of C3/EZB DLBCLs and the incidence of mutations in epigenetic modifier genes are more frequent in FL than in DLBCLs (mutant *KMT2D,* >60%; mutant *CREBBP*, >50%; mutant *EZH2*, >15%) (Okosun et al., 2014; Pasqualucci et al., 2014; Green et al., 2015; Krysiak et al., 2017). Similar to C3/EZB DLBCLs, acquisition of *MYC* translocation, amplification or activating mutations is associated with aggressive histology in FL, predisposing to transformation to aggressive DLBCL (Pasqualucci et al., 2014). Thus, mutational characteristics of GCB-DLBCL and FL are similar and mutations in epigenetic modifier genes may play essential roles for the development and progression of these diseases.

## Metabolic Features of Normal and Tumor GC B Cells

GC B cells, especially in the DZ, are highly proliferative and need to activate specific transduction programs to meet high energetic and biosynthetic demands. These dynamic processes are possible thanks to the great metabolic plasticity of B cells in the GC. Naive B cells are metabolically quiescent and require low levels of catabolic metabolism to sustain energy homeostasis. Following activation, B cells re-shape their metabolic program to meet the energetic and biosynthetic demands for proliferation (Jellusova, 2020). GC B cells use different carbon energy sources and metabolic pathways depending on their stage in the GC reaction process (Choi and Morel, 2020). Studies in mice showed that in comparison with naïve/resting B cells, GC B cells upregulate glucose consumption, together with upregulation of gene signatures for glycolysis, TCA (tricarboxylic acid) cycle and OxPhos (oxidative phosphorylation). These cells present increases in mitochondrial mass and HIF-1α accumulation (Jellusova et al., 2017; Jellusova and Rickert, 2017). Inhibition of glycolysis with hexokinase inhibitors or 2-deoxy-D-glucose (2-DG) significantly decreases the percentage of GC B cells, without affecting the overall percentage of B cells, CD4^+^PD1^+^ T_FH_ cells or the CD4^+^:CD8^+^ T-cell ratio in GCs, pointing to the preferential dependency of GC B cells on glycolysis (Jellusova et al., 2017). However, without detailed direct metabolic analyses *in vivo*, the specific metabolic demand and related metabolic pathways of GC B cells are difficult to precisely determine. Studying these parameters *in vitro* is limiting and has led to conflicting results. For example, MS (mass spectroscopy) of spleen-derived CD19^+^B220^+^CD4^−^CD8^−^ B cells cultured in ^13^C6-glucose detected reductions in total glycolytic metabolites, except for 3-phosphoglycerate (3-PG) upon B-cell activation. Moreover, lactate level accumulation was not observed in these studies (Waters et al., 2018). The isotopologue distribution in glycolytic metabolites suggested that glucose fluxed through the glycolytic pathway without accumulation of lactate, probably routed into alternative metabolic pathways (Waters et al., 2018). Recently, Weisel et al.(Weisel et al., 2020), using freshly isolated primary GC B cells, have shown that these cells are poorly glycolytic and consume higher oxygen amounts than resting naïve B cells or activated T cells. Specifically, GC B cells were found to oxidize both endogenous and exogenous fatty acids through high expression of the fatty-acid transporter CD36 (Weisel et al., 2020). These studies highlight a complex relationship between metabolic and activation states of B cells during the GC response. Interestingly, the transcriptional repressor Bcl6, which deeply controls the GC B-cell program, was identified among the genes specifically regulated in adipocytes, suggesting a role for BCL6 in lipid metabolism. Correspondingly, Bcl6-deficient mice were found to exhibit multiple features of dysregulated lipid metabolism (LaPensee et al., 2014).

According to the described metabolic features of normal GC B cells, a bioinformatics study in DLBCLs revealed that 30% of these tumors rely on OxPhos. In this work, DLBCLs were divided into three subgroups, based on COO and genetic basis for transformation: 1) OxPhos, 2) B-cell receptor (BCR)/proliferation, and 3) “host response” (HR) subsets (Monti et al., 2005). In comparison with BCR-DLBCLs, OxPhos-DLBCLs were found to display enhanced mitochondrial energy transduction, greater incorporation of nutrient-derived carbons into the TCA cycle, and increased glutathione levels. Moreover, perturbation of the fatty acid oxidation (FAO) program and glutathione synthesis proved selectively toxic to this tumor subset, providing evidence for distinct metabolic dependencies and underlying pro-survival mechanisms in DLBCLs (Caro et al., 2012). The differential utilization of fatty-acid-derived carbons and glucose in OxPhos vs. non-OxPhos DLBCLs correlated with the absence or presence of functional BCR signaling, respectively, presenting an example of heterogeneity in nutrient use within the same disease entity. Besides, the utilization of palmitate-derived acetyl-CoA for ATP production and citrate synthesis in OxPhos-DLBCLs suggests that FAO and fatty acid synthesis may coincide in these cells and the inhibition of the mitochondrial FAO program can compromise the survival of OxPhos-DLBCLs. Notably, OxPhos-DLBCLs have shown specific resistance to pan-HDAC inhibitors (HDACis) linked to upregulation of antioxidant pathways after HDAC inhibition, indicating that lymphoma metabolic subtypes may predispose to differential responses to epigenetic therapies (Mensah et al., 2021).

DLBCLs are also considerably dependent on the mitochondrial lysine deacetylase enzyme SIRT3, which belongs to the NAD^+^ dependent deacetylase family sirtuins and regulates anaplerotic glutaminolysis to fuel the TCA cycle and ensures elevated production of biosynthetic precursors needed for rapidly growing lymphoma cells (Li M. et al., 2019). In addition, SIRT3 promotes mitochondrial metabolism and reduces reactive oxygen species via multiple mechanisms. Interestingly, reliance on SIRT3 in DLBCL is independent of the COO (Alizadeh et al., 2000) or the OxPhos or BCR categories (Monti et al., 2005).

## Principles Linking Metabolism to Epigenetics

**FIGURE 1:**
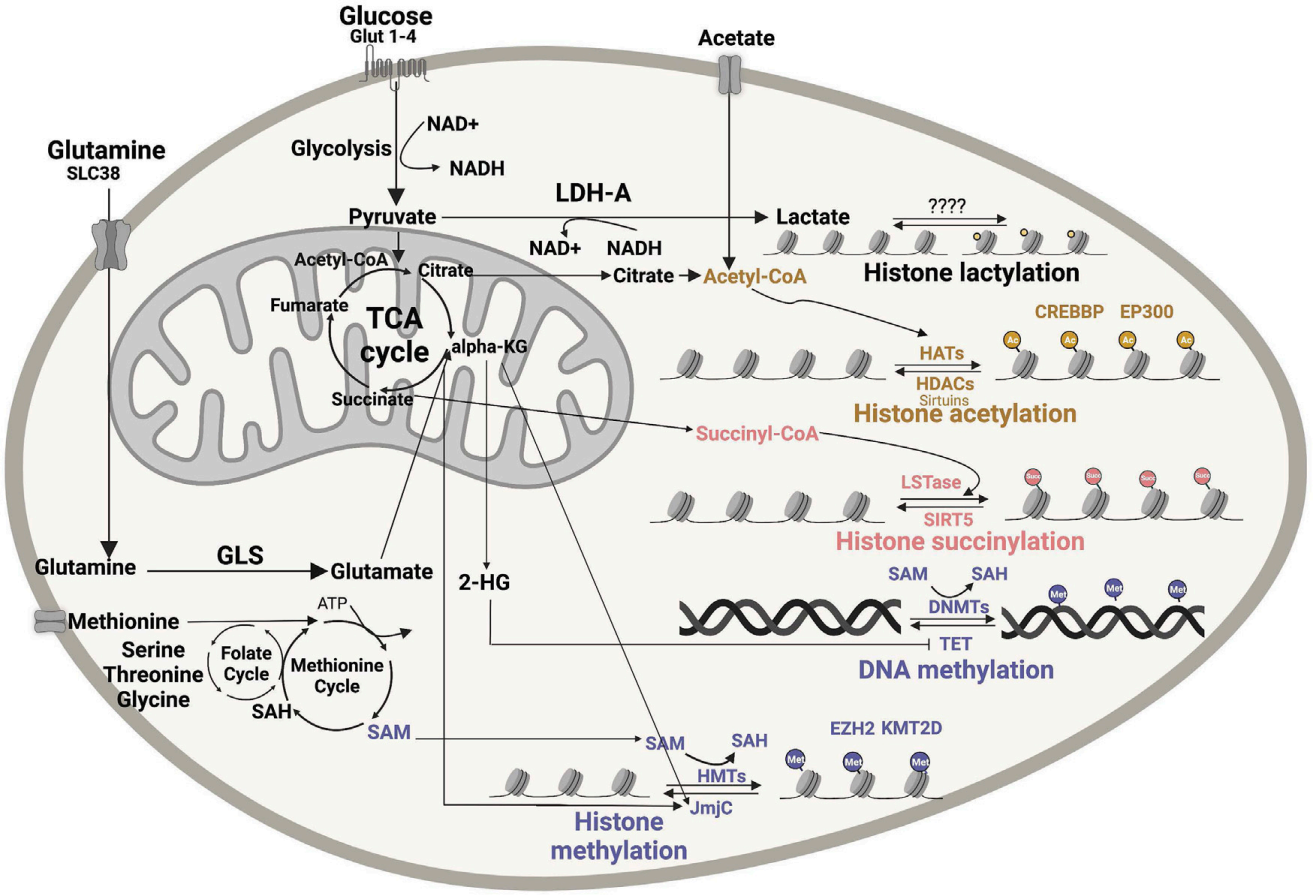
Impact of metabolic intermediates on cellular epigenomic. Numerous nutrients are metabolized to produce intermediates that can be used as substrates or modulators of enzymes involved in chromatin remodeling. Acetyl-CoA, lactate, succinyl-CoA and SAM are the major metabolic bioproducts involved in epigenetic reactions, including histone acetylation, histone methylation, succinylation, lactylation and DNA methylation. DNMT, DNA methyltransferases; GLS, glutaminase; 2-HG, 2-hydroxyglutarate; a-KG, α-ketoglutarate; SAH, S-adenosyl homocysteine; SAM, S-adenosyl methionine dehydrogenase 1; HAT, histone acetyltransferase; HDAC, histone deacetylases; HMT, histone methyltransferase; JmjC, Jumonji N/C-terminal domains; LDH-A, lactate dehydrogenase A; LSTase, lysine succinyltransferase; NAM, nicotinamide; SIRT, sirtuin; TCA, tricarboxylic acid; EZH2- Enhancer Of Zeste 2 Polycomb Repressive Complex 2 Subunit, KMT2D- Histone-lysine N-methyltransferase 2D. Figures were created using BioRender.com.

The activity of epigenetic modifiers is influenced by cellular metabolism and the availability of metabolic products, which in tumors can depend on oncogenic alterations and/or on the tumor microenvironment (TME) (Table 1). DNA and histone methyltransferase and demethylases, histone acetyltransferases (HATs), and deacetylases (HDACs) utilize as substrates and co-factors metabolites derived from serine-glycine-one carbon metabolism, methionine, TCA cycle, ß-oxidation, glycolysis, and hexosamine biosynthesis (Allis and Jenuwein, 2016; Greer and Shi, 2012). More specifically, S-adenosyl methionine (SAM) from the one-carbon metabolism pathway, acetyl-coenzyme A (acetyl-CoA) from TCA-derived citrate, NAD+ from glycolysis or electron transport chain, α-ketoglutarate (αKG) from the TCA cycle, uridine diphosphate N-acetylglucosmaine and other metabolic intermediates in these pathways serve as substrates for chromatin-modifying enzymes (Figure 1). Acetyl-CoA, which is the donor for histone acetylation reactions, and SAM, which is the universal donor for all epigenetic methylation reactions involving DNA and histones, are generated through glucose, amino acid, fatty acid and vitamin metabolism are rate limiting substrates for these chromatin modifying reactions (Kim and Costello, 2017; Su et al., 2016). Therefore, these epigenetic modifications depend on the availability of substrates derived from specific metabolic pathways (Figure 1). For example, enzymes involved in histone and DNA methylation and demethylation can be regulated by both methionine metabolism (generating SAM) and TCA cycle (generating αKG as co-substrate for JmjC histone demethylases), thus linking epigenomic changes to the metabolic state of a cell (Figure 1). DNA methylation alters chromatin structure and regulates gene expressions by converting cytosine into 5-methylcytosine (5 mC). Changes in histone methylation at lysine (K) or arginine (R) amino acid residues can either activate or repress transcription. Histone methylation status can range from mono-/di-/tri-methylation or full demethylation, creating a diverse array of methylation patterns. Both histone and DNA methylation require SAM as the high-energy methyl donor, preferably localized in the nucleus (Figure 1). Acetyl-CoA, which is synthetized from glucose oxidation to pyruvate through pyruvate dehydrogenase (PDH), or from fatty acid ß-oxidation and acetate, metabolically sustains ATP production under aerobic condition and several biosynthetic processes. When not needed for these downstream metabolic processes, acetyl-CoA can diffuse from cytoplasm to the nucleus or can be locally produced in the nucleus (Nitsch et al., 2021), thus becoming available as substrate for HATs to modify histone tails (Figure 1), which is one of the major determinants of chromatin epigenetic state impacting on gene expression.

**TABLE 1:** Main metabolic substrates and co-factors of epigenetic enzymes.

Epigenetic reactions	Metabolites as cofactors and regulators of epigenetic enzymes	Mechanism and examples
Histone and DNA methylation	SAM, SAH (methionine cycle), FAD, α-KG (TCA cycle), succinate (TCA cycle), fumarate (TCA cycle), 2-HG (TCA cycle)	Methyl donors for methyltransferases
		Cofactors for α-KG-utilizing dioxygenases
		Positive regulators of LSD1 and LSD2 (lysine-specific histone demethylase)
		Inhibition of α- KG-utilizing dioxygenases
Histone acetylation	acetyl-CoA (TCA cycle, acetate), NAD+, NAM, ß-hydroxybutyrate	Acetyl donors for acetyltransferases
		Activation of histone deacetylase (SIRT) and PARP
	butyrate, succinyl-CoA (TCA cycle)	Inhibition of histone deacetylase, histone succinylation

Other metabolic intermediates, such as S-adenosylhomo-cysteine (SAH) and ß-hydroxyglutarate (2-HG), can instead affect the activity of chromatin-modifying enzymes by competitively inhibiting SAM and αKG substrate utilization, respectively. Notably, when not efficiently utilized or too abundant in the cell, SAM can regulate the folate cycle by directing it away from sustaining the methionine cycle, thus lowering SAM levels themselves. Overall, these observations indicate that the activity of epigenetic enzymes must tightly respond to changes in cellular metabolism (Reid et al., 2017).

Despite multiple studies suggesting a link between cellular metabolism and histone modifications, the integration of metabolic signals into chromatin changes via histone methylation and acetylation is challenging. Recent progress in MS and metabolic tracing approaches is now deepening our understanding of these mechanisms. By MS, it was recently found that histone modifications—especially acetylation—can be regulated both enzymatically and nonenzymatically (Simithy et al., 2017). Using metabolic tracing of [^13^C3] lactate, Zhang et al.(Zhang et al., 2019) identified lactylation as a new histone modification derived from lactate (Figure 1). Mentch et al.(Mentch et al., 2015) provided evidence that both SAM levels and the SAM/SAH ratio can be quantitatively altered through changes in the metabolic flux of the methionine cycle to affect a chromatin status. Overall, MS is proving an extremely useful tool to dissect the impact of metabolic pathways on epigenetic modifications (Lu et al., 2020). Similar technologies and assays applied to B-cell lymphomas that heavily rely on epigenetic reprogramming will provide new insights into the regulation of epigenetics through cellular metabolism in these diseases, with the potential to unveil novel vulnerabilities that can be targeted for therapy.

## Role of the Immune Microenvironment in GCB Lymphomas

**FIGURE 2:**
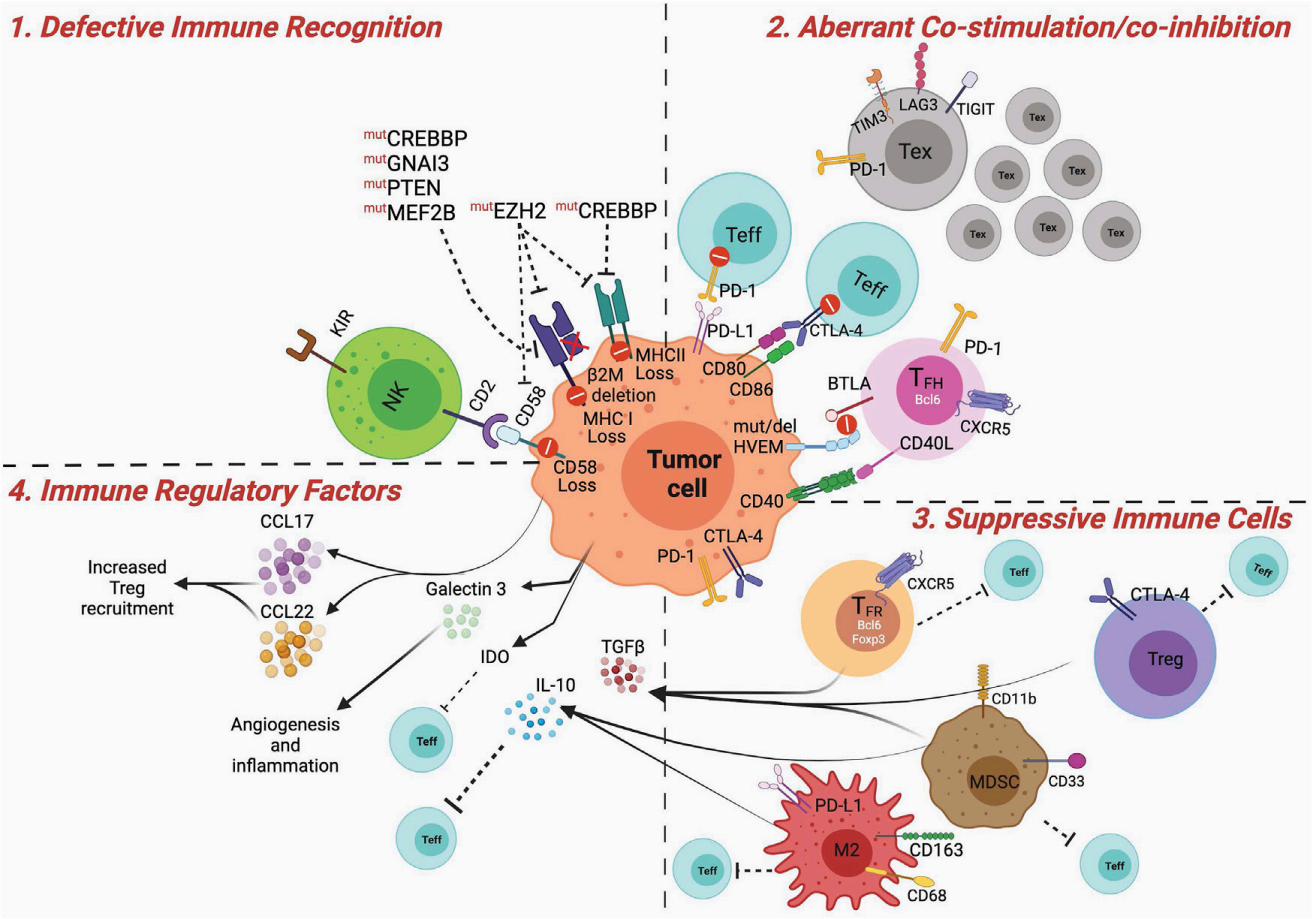
Dysfunctional immune microenvironment in GCB Lymphomas. Major mechanisms contributing to a dysfunctional and suppressive immune microenvironment in GCB lymphomas. 1) Defective immune recognition: MHC-I and MHC-II expression are often downregulated in GCB lymphoma cells, via mechanisms involving various genetic and epigenetic mutations, leading to poor antigen recognition. In addition, disruption of CD58/CD2 axis impedes tumor recognition by NK cells. 2) Aberrant co-stimulation: PD-1 and CTLA-4 can be expressed on tumor-infiltrating effector T cells (Teff) limiting or counteracting their activation via signals received by PD-L1 or inhibition of CD80/CD86 mediated co-stimulation. Tumor B cells can directly express PD-1 and CTLA-4 contributing to dampening T-cell activation. Mutation or deletion of HVEM on the lymphoma cells, renders them non-reactive to BTLA expressing T_FH_ cells, and leads to aberrant expansion of lymphomagenic population (see text for more details). Lymphogenic B cells can proliferate independent of CD40/40L-mediated T_FH_ cell help. Tumors are infiltrated by large numbers of exhausted T cells that, expressing TIM3, LAG3 and TIGIT, are subjected to sub-optimal co-stimulation and activation. 3) Suppressive immune cells: Tregs, T_FR_, M2 macrophages and MDSCs suppress activation of Teff cells. This can be mediated by receptor ligand interaction, such as PD-1:PD-L1, CTLA-4:CD80/CD86 or via soluble factors. 4) Immune regulatory factors: IL-10, TGFb, IDO secreted by MDSCs, macrophages, Tregs, T_FR_, or tumor cells induce immune suppression, by impeding optimal DC priming, promoting M2 polarization, or Treg differentiation. In addition, the release of chemoattractant (e.g. CCL17, CCL22) for suppressive immune cells can further contribute to the establishment of an immune suppressed lymphoma microenvironment. Figures were created using BioRender.com.

The role of the immune microenvironment in B-cell lymphoma pathogenesis is well recognized (Dave et al., 2004; Kotlov et al., 2021); however, the relative impact of different immune cell types on lymphoma immune escape and immunotherapy resistance is not entirely clear. For example, except for CD8^+^ T cells that seem to associate with better prognosis in B-NHL patients (Álvaro et al., 2006; Chang et al., 2007; Wahlin et al., 2007; Wahlin et al., 2010), other T-cell subsets, such as immunosuppressive regulatory T cells (Tregs) and T cells expressing various immune checkpoints variably associate with either positive or negative outcomes (Ansell et al., 2001; Carreras et al., 2006; Tzankov et al., 2008; Carreras et al., 2009; Farinha et al., 2010; Wahlin et al., 2010; Rajnai et al., 2012; Yang et al., 2012; Keane et al., 2013; Brady et al., 2014; Coutinho et al., 2015; Yang et al., 2015; Zhou et al., 2017; Greenbaum et al., 2019). B-cell lymphomas are unique given the fact that these tumor cells arise from professional antigen presenting cells (APCs)—a specialized subset of immune cells able to capture and optimally present antigens (Ags) to T cells through both MHC (major histocompatibility complex)-I and MHC-II routes (de Charette et al., 2016). This would suggest that these tumors are highly immunogenic in nature and may need to induce specific mechanisms of immune suppression to evade immunosurveillance. Aberrant oncogene expression in B cells can occur via genetic alterations during Ig gene rearrangements, but these potentially lymphomagenic cells are often recognized and eliminated by the immune system (Upadhyay et al., 2015). Nonetheless, depending on the sets of oncogenic mutations accumulated over time in altered B cells and their impact on direct pro-survival signals and immune evasion, the tumor can eventually manifest, indicating complete escape from the immune system. In this section we discuss the most common mechanisms of immune dysfunction and immunosuppression observed in GCB lymphomas, including DLBCLs and FLs (Figure 2).

### Defective Antigen Presentation

Downregulation or complete loss of Ag presentation machinery is observed in DLBCL (Challa-Malladi et al., 2011; Nijland et al., 2017) and MHC-I loss has been reported in 40–60% of DLBCL cases (Challa-Malladi et al., 2011; Rimsza et al., 2006; Rimsza et al., 2004). Genetic mutations or loss of beta2-microglobulin (β2M), which destabilize the assembly of the MHC class I, is a primary cause for MHC-I downregulation (Figure 2). β2M mutations are observed in 29% of DLBCL cases (Challa-Malladi et al., 2011)*,* and cytoplasmic β2M isoforms were detected in 48.4% MHC-I-negative DLBCL cases (Nijland et al., 2017). In FL, ∼20% cases harbor MHC-I mutations (Fangazio et al., 2021) and β2M genetic alterations are generally rare (Green et al., 2015), but their frequency increases post histological transformation (Pasqualucci et al., 2014). Mutations in EZH2, GNA13, and MEF2B, as well as PTEN deletions, are significantly associated with MHC-I loss in DLBCLs (Figure 2) (Ennishi et al., 2019b). As discussed below in more detail, EZH2 activating mutations contribute to both MHC-I and MHC-II repression (Ennishi et al., 2019b). Around 40–50% of DLBCL cases have low MHC-II expression, which correlates with poor lymphocytes infiltration and shorter survival in DLBCL patients (Rosenwald et al., 2002; Roberts et al., 2006). MHC-II expression is under stringent epigenetic regulation. CREBBP activates the MHC-II-gene-expression regulator CIITA by catalyzing promoter/enhancer H3K27Ac. CREBBP LOF mutations prevent CIITA transcription in FLs and DLBCLs. MHC-II downregulation in DLBCL also results from overexpression of the TF FOXP1, which seems to be independent of CIITA mutation (Brown et al., 2016). Genetic alterations on chromosome 3p leading to FOXP1 overexpression are found in a small subset of DLBCLs (Koon et al., 2007). FOXP1 translocations are rare in DLBCLs and are often associated with extra-nodal localizations and high proliferative index (Haralambieva et al., 2006). MHC-II expression is reduced in FL cells at both transcription and protein levels, resulting in impaired Ag presentation (Green et al., 2015; Andor et al., 2019). Pharmacologic inhibition of EZH2 or thymidylate synthase (TS) were found to enhance MHC-I expression in human DLBCL cell lines (Dersh et al., 2021). TS contributes to the biosynthesis of thymidine and is important for DNA replication and repair. Interestingly, combined inhibition of EZH2 and TS displayed increased efficacy against DLBCL cells that are resistant to EZH2 inhibitors (Dersh et al., 2021).

### Aberrant Immune Co-stimulation and Co-inhibition

Effective priming of T cells needs two signals from APCs: 1) recognition of the MHC-Ag complex by the T-cell receptor (TCR) and 2) co-stimulation by the interaction between CD80/CD86 on APCs and CD28 on T cells. CD80 and CD86 are B7 family members and are two of the most important mediators of this second signal post Ag recognition. Additional ligands for immune co-stimulatory and co-inhibitory receptors on T cells further fine tune T-cell activation and fate (Chen and Flies, 2013). In this regard, two major examples are CTLA-4 (cytotoxic T-lymphocyte associated protein 4)—a T-cell co-inhibitory receptor with higher affinity for the same CD28 ligands CD80/CD86 (Lee et al., 1998), and PD-1 (programmed cell death protein 1)—another crucial T-cell co-inhibitory receptor dampening the TCR and CD28 downstream signaling pathways upon engagement with its ligands PD-L1 and PD-L2, which can be expressed by tumor cells and immune cells, including APCs (Figure 2) (Pauken et al., 2021).

The role of CD80/CD86 is not yet fully clear in lymphoma, but their expression has been noted on tumor cells and/or on cells from the TME (Greaves and Gribben, 2013). 97% of FL cases and approximately 90% of DLBCL cases express CD80 (Dakappagari et al., 2012). Downregulation of CD80/CD86 has been associated with poor T-cell infiltration in DLBCL (Stopeck et al., 2000). As CD80 can directly interact with PD-L1 with either immune stimulatory or inhibitory outcomes (Pauken et al., 2021), the hierarchy and all possibility of these interactions in the various lymphoma TME need to be considered when assigning patients to ICB-based treatments.

CTLA-4 expression is detectable in both DLBCL and FL cells. Using human lymphoma cell line OCI-Ly3, Hermann et al. have shown that CTLA-4 expressed on these cells can interact with soluble CD86 and internalize it (Herrmann et al., 2017). This interaction can trigger the STAT3 (signal transducer and activator of transcription 3) pathway via phosphorylation of Tyk2 (tyrosin kinase 2) in B cells, with consequent induction of the immunoregulatory cytokines IL-10 and IL-6 (Herrmann et al., 2017), which facilitates immune evasion and supports tumor growth. In the same study, using a co-culture system, the authors showed that A20 lymphoma cells can internalize CD86 expressed on APCs via CTLA-4.

PD-1 is constitutively expressed on naïve B cells and is rapidly recruited to the immune synapse with BCR upon B-cell activation (Thibult et al., 2013). Various structural chromosomal alterations, including translocations involving the Ig heavy chain (IgH) locus or disruption of the 3’ region of the PD-L1 gene, can lead to aberrant PD-L1 expression in DLBCL (Georgiou et al., 2014; Kataoka et al., 2016). Interestingly, the Ig locus and CIITA are common partners of PD-L1 translocations in DLBCL (Steidl et al., 2011; Chapuy et al., 2016; Georgiou et al., 2016). However, the frequency of such events in GCB lymphomas is overall low, which may explain at least in part the lack of activity of PD-(L)-1 blockade in these diseases. In FL, a small fraction of neoplastic B cells (∼5%) and histiocytes express PD-L1 (Carreras et al., 2009; Myklebust et al., 2013).

T cells infiltrating lymphoma tissues compared to tonsil more frequently express PD-1 and display an exhausted phenotype, including co-expression of other T-cell inhibitory receptors, such as TIM3 (T-cell immunoglobulin (Ig) and mucin-domain-containing molecule 3) in FL (Yang et al., 2017), LAG3 (lymphocyte activating 3) in DLBCL (Roussel et al., 2021) and/or TIGIT (T-cell immunoreceptor with immunoglobulin and ITIM domains) in both FL and DLBCL (Figure 2) (Josefsson et al., 2019). PD-1 expression is detected in the TME of 39.5–68.6% of DLBCL cases (Song et al., 2019), but it has mixed clinical implications. While some studies observed that PD-1^+^ tumor-infiltrating lymphocytes (TILs) are associated with favorable clinical outcome in DLBCL (Muenst et al., 2010; Ahearne et al., 2014; Kiyasu et al., 2015; Fang et al., 2017), a recent study by Enemark et al. showed that PD-1 on intrafollicular T cell is a predictive biomarker for histological transformation of FL into DLBCL (Beck Enemark et al., 2021). In DLBCL, PD1^+^TIM3^+^CD8^+^T cells with an effector memory phenotype are observed inside CD20^+^ B-cell clusters (Roussel et al., 2021). In FL, TIM3 is expressed in ∼30–40% TILs (mainly CD8), with even greater expression in functionally exhausted PD-1^low^ T-cell subsets (Yang et al., 2015). A recent study in two independent DLBCL cohorts uncovered similar trends in TIM3^+^LAG3^+^ TIL abundance in these diseases, and these cells were found to be an independent predictor of poor survival (Autio et al., 2021). Consistently, in FL patients, the presence of CD3^+^LAG-3^+^ as well as TIM-3^+^LAG-3^+^ TILs correlates with poor survival (Yang et al., 2017). TIM3 and LAG3 have also found to be expressed on DLBCL cells themselves and high TIM3 expression in these tumor cells correlates with shorter survival in patients (Chen et al., 2019; Keane et al., 2020). Interestingly, in FL patients, exhausted-phenotype TIGIT^+^CD8^+^ T cells and highly suppressive TIGIT^+^ Tregs in the TME contribute to resistance to PD-1 blockade (Josefsson et al., 2018; Yang et al., 2020). While the TIGIT inhibitory ligand CD155 can be expressed in normal B cells, its expression in lymphoma B cells in associating with infiltrating TIGIT^+^ T cells has not been deeply investigated.

The HVEM(TNFRSF14):LIGHT/BTLA/CD160 axis is another relevant immune modulatory pathway in B-cell lymphoma. HVEM can deliver co-stimulatory or inhibitory signals depending on the interactions with LIGHT vs. BTLA or CD160, respectively. While HVEM is mostly expressed in T cells, it can be also found on B cells, and is mutated in a fraction of FLs and often lost in EZB DLBCLs (Mlynarczyk et al., 2019) and other subtypes of DLBCLs (Kennedy and Klein, 2019). This suggests that the HVEM pathway normally controls GC B cells. This effect appears to be mainly mediated by T_FH_, which express high levels of the inhibitory counter-receptor BTLA (B- and T-lymphocyte attenuator) (Figure 2) (Kashiwakuma et al., 2010). T_FH_ cells can have a dual effect on lymphoma B cells, especially of GC origin, because of their physiologic function to provide pro-survival signals (e.g. via CD40L:CD40) only to B cells that have optimally re-arranged their Ig genes and to restrain the growth of the other suboptimal clones (Basso et al., 2004; Good-Jacobson et al., 2010). Loss of HVEM in GCB lymphoma cells inactivates a major mechanism through which T_FH_ properly dose and direct their helper functions toward the most fit B cells. Two complementary preclinical studies showed that HVEM insufficiency in lymphoma cells increases the proportion of T_FH_ and FDCs in the TME (Boice et al., 2016) while concurrently reducing the ability of tumor B cells to interact with T_FH_ (Mintz et al., 2019), leading to overgrowth of HVEM^−/−^ malignant cells. Increases in intratumor T_FH_ that no longer control the HVEM-defective lymphoma clone can in turn support tumor growth through the secretion of IL-4 and other pro-lymphomagenic cytokines (Boice et al., 2016). In this context, ICOS^+^PD-1^+^ T_FH_ cells and their signature cytokine IL-21 are associated with poor therapeutic outcome in B-NHLs (Brady et al., 2014). Interestingly, FL is also enriched in follicular regulatory T cells (T_FR_), which persist along with T_FH_ cells after rituximab therapy and contribute to immune suppression and poor therapeutic outcome (Ochando and Braza, 2017). Mechanistically, it has been shown that mesenchymal stromal cells can induce Foxp3 in FL-associated T_FH_ cells, converting them into T_FR_ *.* Clarifying the biologic significance of the T_FH_/T_FR_ plasticity and potential inter-differentiation in GCB lymphomas will be crucial to understand how to precisely target or repolarize them for improved lymphoma control.

Overall, these findings illustrate the complex rewiring of the immune interactions between lymphoma B cells and T cells through aberrant co-stimulation and co-inhibitory pathways, which contributes to dampening anti-tumor immunity and to lymphoma growth. Clarifying whether and how defined sets of driver mutations in lymphoma specifically reshape the immune microenvironment will add important information to the molecular classification of these diseases and for improved patients’ treatment with immunotherapy. This is particularly relevant for GCB lymphomas, where mutations in epigenetic modifiers have a direct impact on Ag presentation and T-cell co-stimulation. Understanding whether these effects directly impact on the differentiation of specific immune microenvironments will be important for the development of more successful precision immune-oncology treatments for these diseases.

### Cellular and Soluble Mediators of Active Immunosuppression

Major immunosuppressive mediators in lymphoma microenvironment include Tregs, tumor associated macrophages (TAM), myeloid derived suppressor cells (MDSCs), immunoregulatory cytokines/chemokines and other soluble factors, such as products of aberrant tumor metabolism (Figure 2).

Foxp3^+^ Tregs can suppress T- and B-cell function. In DLBCL tissue compared to normal lymph nodes, the frequency of intra-tumoral Tregs increases ∼3 times (38%, vs. 12% of CD4 cells) (Mittal et al., 2008). The prognostic implication of Tregs in B-cell lymphomas is still not entirely clear. In FL, Foxp3^+^ cells measured by immunohistochemistry have been found to correlate with prognosis depending on their spatial distribution, with intrafollicular localization of Tregs being associated with poor survival and risk of transformation (Farinha et al., 2010). More recently, it was shown that the TCR repertoire of the Tregs and CD8^+^ T cells inversely correlated, suggesting an antigen specific suppression of CD8^+^ T-cell clonal expansion (Liu et al., 2015). In other studies in both DLBCLs (Farinha et al., 2010) and FLs (de Charette et al., 2016), instead, Foxp3^+^ cells have been found to be associated with improved outcome after chemotherapy with or without rituximab. This may be attributed to the possibility that Tregs can directly suppress lymphomagenic B cells. More direct functional studies are needed in this area, especially to refine the identity of Foxp3 cells that correlate with outcome in these patients.

TAMs can be grouped in two major categories: 1) CD163^-^ M1 macrophages, which are pro-inflammatory and 2) CD163^+^ M2 macrophages that are anti-inflammatory and are preferentially recruited at the tumor site (Figure 2). In DLBCL, CD68^+^CD163^+^ M2 macrophages are associated with poor clinical outcome (Komohara et al., 2015; Guo et al., 2016) and more frequent extra-nodal involvement (Li Y.-L. et al., 2019). The predictive value of the macrophage marker CD68 alone in lymphoma is debatable, with some studies indicating no predictive value in DLBCL (Matsuki et al., 2019), and others observing correlation with poor prognosis (Cai et al., 2012; Nam et al., 2014; Riihijarvi et al., 2015). Intriguingly, TAMs in DLBCL show STAT3-mediated expression of PD-L1 and this signature was found to correlate with prolonged PFS (McCord et al., 2019). The prognostic impact of TAMs in FL is not fully clear and can depend on the type of treatment. In an early study with FL patients treated with combination chemotherapy followed by radiation, elevated TAMs predicted inferior survival (Farinha et al., 2005). However, these cells did not correlate with poor survival if the patients received rituximab (Taskinen et al., 2007). The GELA FL-2000 clinical trial also showed that high frequency of intra-tumoral macrophages correlated with poor survival only in the patients who received chemotherapy without rituximab (Canioni et al., 2008). To incorporate the microenvironment component in the prognosis algorithms, the Lunenburg Lymphoma Biomarker Consortium studied a homogeneously rituximab-chemotherapy-treated group of FL patients and found that low CD8^+^ T-cell percentages, the presence of CD163-expressing macrophages, *EZH2* wild-type (WT) status and gain of chromosome 18 in the diagnostic tumor biopsies predict a poor prognosis in FL treated with R-CHOP (Stevens et al., 2017), pointing to an overall negative impact of macrophages in the outcome of FL patients in these conditions.

MDSCs can be divided into two groups: polymorphonuclear (PMN-MDSC) and monocytic (M-MDSC) (Zhou et al., 2018; Tcyganov et al., 2018). Typically, in humans, MDSCs are identified by myeloid cell markers CD11b^+^, CD33^+^, HLA-DR low/−, and lineage-specific antigen Lin-negative (Figure 2). MDSCs can attenuate anti-tumor cytotoxic T lymphocyte (CTL) responses via metabolic competition, and generation of oxidative stress (Gabrilovich et al., 2012). A recent study by Wang et al. described a higher proportion of functionally suppressive M-MDSCs in DLBCL patients, which correlated with disease stage (Wang Z. et al., 2021). In an earlier preclinical study using the A20 lymphoma model, the authors showed that MDSCs can activate Tregs, thus reinforcing local immunosuppression (Serafini et al., 2008). Whether this mechanism occurs in human lymphoma remains to be established.

MDSCs, M2 macrophages and Tregs can secrete IL-10 (Shen et al., 2016), which inhibits T-cell function, contributing to a suppressive lymphoma microenvironment (Figure 2). Another mechanism through which immunoregulatory myeloid cells limit T-cell function is by depleting critical nutrients for activated, proliferating T cells which are metabolically demanding. Tryptophan is an essential amino acid, critical for T-cell function, which can be degraded by indoleamine 1,2,3-dioxygenase (IDO). IDO is overexpressed in MDSCs and was also found to be upregulated in lymphoma cells (Elpek et al., 2007). It has been shown that intrasplenic injection of lymphoma cells in mice leads to Treg recruitment and that this effect is counteracted by IDO1 inhibition (Curti et al., 2007). TGFβ is another well-established immunosuppressive cytokine responsible for suppression of CD8^+^ effector T cells in the TME (Figure 2). However, the role of TGFβ in lymphoma is debated, as studies suggest that activation of this pathway might confer survival advantage to both DLBCL (Merdan et al., 2021) and FL patients (Labidi et al., 2010). In addition, in FL patients, elevated serum levels of IL-1R1, IL-6, IL-7, IL-10, IL-13, TNF-α, and vascular endothelial growth factor (VEGF) were identified (Labidi et al., 2010). Elevated serum VEGF and the glycolytic enzyme lactate dehydrogenase (LDH) are associated with shorter PFS in FL (Labidi et al., 2010). Locally in the TME, it was shown that T_FH_ can induce FL cells to release the chemokines CCL17 and CCL22, which can in turn recruit Tregs and more IL-4-producing T cells to sustain tumor growth and immunosuppression (Rawal et al., 2013). Galectin 3 is another relevant soluble factor that can contribute to local immunosuppression in DLBCL and FL. A study by D'Haene et al. showed that galectin-3 is expressed in 50% of the DLBCL cases and 12.5% of FL cases (D'Haene et al., 2005). Galectin-3 mediates pro-tumor inflammatory process and is important in recruitment of macrophage and angiogenesis—which could potentially contribute to immune evasion collectively.

## Impact of Major Epigenetic Modifiers in GCB Lymphoma Metabolism and Immune Evasion

Critical epigenetic modifiers commonly altered in GCB-DLBCL and FL modulate the way B cells interact with immune cells in GCs and require metabolic substrates as co-factors, pointing to a key role of these alterations in immune evasion of GCB lymphomas, which may be supported by specific metabolic processes (Figure 2).

### EZH2

EZH2 is responsible for the enzymatic activity of polycomb repressive complex 2 (PRC2) which catalyzes histone 3 lysine 27 trimethylation (H3K27me3) at gene promoters and represses target gene expression (Di Croce and Helin, 2013). EZH2 is essential to maintain GC reactions by inhibiting PC differentiation and cell-cycle checkpoint genes in cooperation with BCL6, and EZH2 loss impairs GC formation (Béguelin et al., 2013; Caganova et al., 2013; Béguelin et al., 2016; Béguelin et al., 2017). Importantly, more than 90% of *EZH2* mutations in DLBCL and FL occur at the Y641 residue located in the catalytic SET domain, which result in the GOF of EZH2 catalytic activity (Morin et al., 2010). Genetically engineered mice to specifically express the *Ezh2* ^*Y641F*^ point mutation in B cells develop GCB-like lymphomas in cooperation with BCL2 or BCL6 overexpression (Béguelin et al., 2013; Béguelin et al., 2016). Since *EZH2* mutations in DLBCL and FL enhance EZH2 catalytic activity and EZH2 is essential for the development of GC B cells, EZH2 targeted therapy is a precision approach against GCB lymphomas. Indeed, pharmacologic inhibition of EZH2 is highly effective for the treatment of murine *Ezh2*-mutant B-cell lymphomas as well as *EZH2*-mutant-patient-derived xenograft (PDX) models *in vivo* (Béguelin et al., 2013; Béguelin et al., 2016; Scholze et al., 2020). As we describe in detail in the last section of this review, tazemetostat is the first FDA-approved EZH2 inhibitor for FL patients, which has shown activity especially in patients with *EZH2-*mutant FL (Morschhauser et al., 2020).

EZH2 methyltransferase activity can be regulated by SAM levels and EZH2 can self-sustain its own methylation activity by promoting SAM synthesis (Dann et al., 2015) (Figure 1). Other metabolites can post-translationally modify EZH2 (phosphorylation, *O*-GlcNAcylation, acetylation, methylation, ubiquitination) leading to function and stability alterations of PRCs (Li et al., 2020). In other type of cancers, EZH2 has been shown to suppress several metabolic activities, including branched amino acid (BCAA) metabolism, TCA cycle (IDH1), mTOR (mammalian target of rapamycin) signaling, and glutamine metabolism (Dann et al., 2015; Gu et al., 2019). EZH2 can also serve as a sensor of glycolytic metabolism in the TME through the miRNA-EZH2-Notch signaling pathway and this pathway is in turn regulated by glucose metabolism in the TME (Zhao et al., 2016). Overall, these observations indicate not only that EZH2 activity impacts on cell metabolism, but also that cell metabolism can influence EZH2 function. These effects deserve precise investigation in lymphoma, especially in *EZH2*-mutant cases.

*EZH2* activating mutations in GCB lymphomas significantly alters the immune microenvironment. *EZH2* ^Y641F^ promotes abnormal expansion of centrocytes in GCs by preventing apoptosis and activation of the MYC pathway, which is crucial for recycling into the DZ (Béguelin et al., 2020). *EZH2* ^Y641F^-mutant centrocytes downregulate T_FH_-interacting molecules such as SLAM, ICAM-1, ICAM-2, and Ly108 and are less dependent on the CD40/CD40L pro-survival signals induced by T_FH_ (Béguelin et al., 2020). This gives a survival advantage to mutant centrocytes in a competitive microenvironment with WT centrocytes. *EZH2* activating mutations in DLBCL patients are also strongly associated with loss of both MHC-I and MHC-II molecules (Ennishi et al., 2019b), which facilitates immune evasion. A recent study using genome-wide CRISPR screening in DLBCL cell lines has identified critical positive and negative regulators of MHC-I expression, among which EZH2 is the most crucial one in GCB-DLBCL (Dersh et al., 2021). Furthermore, EZH2 GOF is closely associated with epigenetic silencing of CD58 expression on lymphoma cells, thus blocking the interaction with cytotoxic effector CD2^+^ T and NK cells and interrupting another avenue of immune control (Figure 2) (Otsuka et al., 2020).

### KMT2D

KMT2D is a part of the COMPASS-like complex which regulates gene enhancer functions through histone 3 lysine 4 mono- and di-methylation (H3K4me) for active gene transcription (Ford and Dingwall, 2015; Froimchuk et al., 2017). Among patients with GCB-DLBCL and FL, most *KMT2D* mutations are frameshift or nonsense mutations which result in KMT2D LOF (Zhang et al., 2015). *Kmt2d*-deficiency impairs B-cell differentiation and induces expansion of GC B cells in mice, suggesting that KMT2D is required to terminate GC reaction and promote PC differentiation (Ortega-Molina et al., 2015; Zhang et al., 2015). *KMT2D* LOF mutations accelerate B-cell lymphomagenesis in cooperation with *Bcl2* in mice (Ortega-Molina et al., 2015; Zhang et al., 2015). KMT2D-target enhancers are repressed by BCL6 during GC reactions through the recruitment of LSD1, a histone demethylase at H3K4 (Hatzi et al., 2013). LSD1 loss in GC B cells impairs GC formation and prevents BCL6-driven lymphomagenesis through de-repression of BCL6 target genes (Hatzi et al., 2019). Although LSD1 knockdown inhibits the proliferation of DLBCL cell lines *in vitro*, pharmacologic inhibition of the enzymatic activity of LSD1 only shows modest effects against DLBCL *in vivo* (Hatzi et al., 2019). Since LSD1 is also responsible to recruit CoREST complex which induces a repressive chromatin state through the activity of HDAC1/2 in the complex, inhibition of the catalytic activity of LSD1 might not be enough to restore the expressions of B-cell differentiation genes (Shi et al., 2005; Yang et al., 2006). Therefore, LSD1 degraders rather than inhibitors of LSD1 enzymatic activity might be suitable for precision therapy of *KMT2D*-mutant GCB-lymphomas. KDM5 is another histone lysine-specific demethylase, which demethylates H3K4me1 to H3K4me0 and H3K4me3/me2 to H3K4me1. Notably, KDM5 inhibition has been shown to alleviate loss of H3K4 activating methylation marks in *KMT2D*-mutant lymphomas and may constitute a viable therapeutic strategy for *KMT2D*-mutant GC lymphomas (Heward J. et al., 2021).

The impact of KMT2D LOF in B-cell lymphoma metabolic rewiring has not been explored yet. However, in lung cancer, where KMT2D was found to be the most highly inactivated epigenetic modifier, *KMT2D*-inactivating mutations induce aberrant metabolic reprogramming via increased expression of glycolytic genes (Alam et al., 2020). Mechanistically, KMT2D was found to upregulate the circadian rhythm repressor PER2 which plays an important role in tumor suppression (Fu et al., 2002). Several glycolytic genes (e.g., *Eno1*, *Pgk1*, *Pgam1*, *Ldha*, *Gapdh*, and *Cdk1*) were identified as target genes of PER2. Therefore, KMT2D-mediated *Per2* activation represents a previously unknown tumor-suppressive mechanism that links an epigenetic tumor suppressor to a circadian rhythm regulator with direct metabolic implications. Accordingly, pharmacologic inhibition of glycolysis reduces tumorigenicity of human lung cancer cells bearing *KMT2D*-inactivating mutations, suggesting that KMT2D deficiency may present a therapeutic vulnerability to glycolytic inhibitors (Ding et al., 2008; Alam et al., 2020). The link between KMT2D and glycolysis may be relevant and worth to explore in detail in lymphoma, as glycolysis measured by expression of aldolase A and GAPDH was associated with significantly shorter transformation-free survival in FL patients (Monrad et al., 2020). High expression of aldolase A and GAPDH may indicate increased metabolic turnover, and these enzymes may be useful biomarkers in primary FL for predicting the risk of subsequent lymphoma transformation. It will be important to determine the extent to which *KMT2D* inactivating mutations support the glycolytic switch in GCB lymphomas.

*KMT2D* LOF mutations are associated with altered immune signatures in DLBCL. In a recent study by You *et al., KMT2D* non-synonymous mutations have been shown to correlate with an overall increase in mutational burden in DLBCL, which intriguingly corresponded with low intra-tumoral T-cell infiltration in GCB DLBCL patients with WT P53 (You et al., 2021). Similarly, in solid cancers, *KMT2D* LOF mutations have been recently found to contribute to DNA damage, increased mutational burden and activation of transposable elements, which in this setting are associated with increased infiltration of effector immune cells, such as CD8 T cells, NK cells and M1 macrophages and decreased infiltration of Tregs and immature macrophages and better response to ICB activity (Wang et al., 2020; Liu et al., 2021). This highlights potential distinct effects of this epigenetic modifier depending on the disease setting, underscoring the importance to study these mechanisms more carefully and specifically for rational design of more effective combination immunotherapies for lymphoma patients.

### CREBBP and EP300

CREBBP catalyzes histone 3 lysine 27 acetylation (H3K27Ac) at enhancers, which activates transcription through the recruitment of DNA-binding TFs and other co-activators (Green, 2018). CREBBP activates PC differentiation genes, such as PRDM1 and IRF4, which are required to terminate the GC reaction (Jiang et al., 2017; Zhang et al., 2017). Mutations of *CREBBP* in DLBCL and FL are concentrated in the acetyltransferase catalytic domain, with hot spot mutations at the R1446 residue (Pasqualucci et al., 2011b). These mutations reduce CREBBP acetyltransferase activity and promote transcriptional repression of target genes (Pasqualucci et al., 2011b). Conditional *Crebbp* loss in B cells induces focal depletion of H3K27Ac at enhancers and accelerates B-cell lymphoma development in cooperation with *Bcl2* in mice (García-Ramírez et al., 2017; Jiang et al., 2017; Zhang et al., 2017). During GC reactions, the enhancers regulated by CREBBP are generally repressed by BCL6 through recruitment of SMRT/NCOR complexes (Hatzi et al., 2013; Jiang et al., 2017). SMRT/NCOR complexes contain HDAC3 which antagonizes the function of CREBBP through H3K27 deacetylation, and HDAC3 loss in GCB-DLBCL cells restores H3K27Ac marks and enhances BCL6-SMRT target gene expression (Hatzi et al., 2013; Jiang et al., 2017; Mondello et al., 2020). These findings suggest that HDAC3 pharmacologic inhibition can be a promising therapeutic strategy for *CREBBP*-mutant GCB lymphomas, promoting GC B-cell exit and differentiation into PCs (Mondello et al., 2020).

In addition, EP300, which is also responsible for H3K27Ac and whose LOF mutations are found in GCB-DLBCL and FL (Cerchietti et al., 2010), partially compensates for the function of CREBBP, and EP300 may be critical for *CREBBP*-deficient B-cell survival (Meyer et al., 2019). Therefore, EP300 targeted therapy may be another precision approach against *CREBBP*-mutated GCB-lymphoma.

CREBBP and EP300 HAT activity may be modulated by the availability of acetyl-CoA substrates deriving from cellular metabolic processes (Figure 1). While poor evidence of these mechanisms is currently available for lymphoma, initial studies in hepatocellular carcinoma suggest that p300/CBP epigenetically induces expression of glycolysis-related enzymes (Cai et al., 2021), which may sustain HAT activity through increased acetyl-CoA levels.

In FL, *CREBBP* mutations are founder events, occur early and contribute to immune escape by downregulating MHC-II expression (Green et al., 2015; García-Ramírez et al., 2017), which is crucial for GC B-cell differentiation (Allen et al., 2007) and tumor-Ag presentation (Khodadoust et al., 2017). In DLBCL, it has been shown that *CREBBP/EP300* mutations are also associated with the recruitment CD68^+^ and CD163^+^ M2 macrophages to the tumor site (Huang et al., 2021). The skewed M2 polarization in *CREBBP/EP300-*mutant DLBCLs was attributed to aberrant regulation of the FBXW7-NOTCH-CCL2/CSF1 axis (Huang et al., 2021). Moreover, *CREBBP/EP300*-mutant DLBCL patients were found to have higher serum levels of the immunosuppressive cytokine IL-10 compared to the pro-inflammatory cytokine IL-1β (Huang et al., 2021), suggesting potential systemic immune suppression in these patients. *CREBBP* mutations were also associated with upregulation of colony stimulating factor 1 (CSF1) and B7H4, both of which are linked to immunosuppressive myeloid cells. These findings suggest that the reprogrammed myeloid compartment in *CREBBP/EP300*-mutant lymphomas can be considered to identify novel therapeutic targets for patients bearing these diseases.

## Emerging Therapies for B-Cell Lymphomas

### EZH2 Inhibitors

Currently tazemetostat is the only the EZH2 inhibitor (EZH2i) FDA-approved for the treatment of FL and remains under investigation in DLBCL. In 2020, tazemetostat was approved for use in patients with *EZH2*-mutant FL who are relapsed or refractory (R/R) following at least 2 prior therapies, and for patients with WT *EZH2* and R/R FL following 2 prior therapies and without other treatment options (Morin et al., 2021), based on phase-I and phase-II results (NCT01897571) showing efficacy in these populations (Italiano et al., 2018). The initial phase-I dose-escalation study included both patients with solid tumors and B-cell lymphomas, including 13 patients with DLBCL and 7 with FL (Italiano et al., 2018). Of these 20 patients, 7 responded and 3 had a complete response (CR). Notable toxicity included grade 3 or greater thrombocytopenia, anemia, hyperbilirubinemia and transaminitis with a significant number of patients experiencing grade 2 fatigue, anorexia, nausea/vomiting, and muscle spasms as well (Italiano et al., 2018). Following the efficacy in DLBCL and FL patients in the phase-I study, the phase-II portion of the trial recruited patients with R/R DLBCL and FL and treated with 800 mg of tazemetostat twice daily. 99 patients with FL were recruited, including 45 patients with *EZH2*-mutant FL and 44 with WT *EZH2*, with median age and prior lines of therapy similar between the two groups (Morschhauser et al., 2020). Overall response rate (ORR) and CR rate were higher in *EZH2*-mutant vs. WT cohort (69 and 13% vs. 35 and 4%, respectively). Responses were observed also among patients with progression of disease within 24 months of last therapy—an important negative prognostic factor in FL, albeit more frequently again in the *EZH2*-mutant vs. WT patient population (64 vs. 25% ORR, respectively) (Morschhauser et al., 2020). However, median PFS and duration of response (DOR) were similar between patients with *EZH2*-mutant vs. WT FL (13.8 and 10.9 vs. 11.1 and 13.0, respectively) (Morschhauser et al., 2020). Overall, these results showed efficacy in both *EZH2*-mutant and WT FL patients, with similar DOR and PFS between the two cohorts, despite higher response rate in *EZH2*-mutant patients, and similar toxicity profile compared to the phase-I trial (Morschhauser et al., 2020). Therefore, tazemetostat represents a valuable option with a reasonable toxicity profile and clinical efficacy for FL patients that are refractory to multiple prior lines, including those with WT *EZH2*. Preliminary results from the DLBCL portion of the trial (patient n = 226; *EZH2* mutant, *n* = 36) were disappointing. ORR to tazemetostat monotherapy was similarly low (17%) in patients with either *EZH2*-mutant or WT tumors (3% CR for *EZH2*-mutant and 9% CR for *EZH2*-WT). Among 69 *EZH2*-WT patients that were treated with tazemetostat plus prednisolone, ORR was just 9%, with 1% CR, and median PFS and DOR not yet reached (Ribrag et al., 2018). Major toxicities included thrombocytopenia, anemia, neutropenia, nausea/vomiting, and fatigue (Ribrag et al., 2018). The variable results with tazemetostat in both *EZH2*-mutant and WT lymphoma patients underscore the need to identify reliable predictors of response that can help 1) allocate to this treatment the patients that are more likely to respond and 2) anticipate resistance. Systematic analysis of epigenetic, immune and metabolic profiles of the tumors that respond in comparison with tumors that do not respond will provide fundamental information in this direction.

While clinical trials have thus far evaluated tazemetostat mainly as a monotherapy in B-cell lymphomas, several rational drug combinations are currently being studied. A phase-I trial of tazemetostat plus R-CHOP in untreated DLBCL was published in 2020, with a phase-II trial planned to add patients with untreated FL (NCT02889523) (Sarkozy et al., 2020). In R/R FL, trials combining tazemetostat with lenalidomide plus rituximab regimen (NCT04224493) or rituximab (NCT04762160) are currently under way. Preclinical studies have also demonstrated synergy between tazemetostat and venetoclax (Bcl2 inhibitor) against DLBCL, with phase-I trials currently in development (Scholze et al., 2020). Due to its effects on the TME and on T cells, tazemetostat has also been proposed as an adjunct to several immunotherapies, which will be discussed in greater detail below. In addition to tazemetostat, other EZH2is are in development for GCB lymphomas. The highly selective EZH2i GSK2816126 showed disappointing results in a phase-I trial (Yap et al., 2019), while others, including CPI-0209 and SHR2554 (NCT04104776, NCT03603951) are still in clinical testing. Dual EZH1/2is are also currently being evaluated, including CPI-1205 (Harb et al., 2018), and valemetostat (NCT04842877). Clarifying the relative contribution of inhibiting EZH1 together with EZH2 in lymphoma remains an important aspect to determine (Yamagishi et al., 2019). Overall, the future appears to be bright for the potential of EZH2is in GCB lymphomas, with clinical efficacy of tazemetostat already apparent and many novel combinations and new agents in development.

### HDAC Inhibitors

HDAC is have been considered as a form of epigenetic therapy, although there is little evidence to suggest that their anti-lymphoma effects are related to epigenetic regulation. Several such agents have been studied in clinical trials (Cao et al., 2018). The first clinically evaluated HDACi was vorinostat—a pan-HDACi that has now been studied in the phase-II setting for both newly diagnosed and R/R DLBCL and FL, both as monotherapy and in combination with other agents. In R/R DLBCL, vorinostat monotherapy proved very disappointing with underwhelming 6% ORR as well as high incidence of grade 3–4 thrombocytopenia and neutropenia (Crump et al., 2008). Vorinostat has also been combined with rituximab, cyclophosphamide, etoposide, and prednisone (R-CVEP) in R/R DLBCL with an ORR of 57% (35% CR) and a median PFS of 9.2 months with high rates of grade 3–4 hematologic toxicities (Smith et al., 2019). In untreated DLBCL, vorinostat was combined with R-CHOP but did not meet a predefined efficacy improvement over standard R-CHOP(Persky et al., 2018). In R/R FL, vorinostat monotherapy has shown efficacy with two phase-II studies demonstrating ORRs of 47% (Kirschbaum et al., 2011) and 49% (Ogura et al., 2014) with high rates of grade 3 and 4 neutropenia and thrombocytopenia in both studies. Another trial combined vorinostat with rituximab in R/R FL demonstrated similar 50% ORR and toxicity profile, with a 2-years PFS of 61% (Chen et al., 2015). Belinostat, another pan-HDACi, has also been studied in R/R DLBCL or transformed FL as monotherapy, but like vorinostat, it showed dismal response rates with just 11% ORR, although toxicity was more manageable than with vorinostat (Puvvada et al., 2016). Mocetinostat is an isotype-specific HDACi that was studied as monotherapy in R/R DLBCL and FL. ORR in both diseases was low at 19% in DLBCL and 12% in FL, with the most common grade 3 and 4 adverse events being neutropenia, thrombocytopenia, and fatigue (Chen et al., 2020). Another pan-HDAC inhibitor abexinostat, with a different pharmacokinetic profile, although showing promising results in FL and DLBCL patients, with ORRs of 56%, and 31% respectively, induced frequent ≥ grade 3 adverse events (Ribrag et al., 2017). Overall, the clinical activity with unselected HDACis have been limited by the pleiotropic and toxic effects, especially hematologic adverse events.

Based on the specific role of HDAC3 in lymphomagenesis, highly specific HDAC3is may offer a better option to both improve clinical efficacy and reduce toxicity from off-target effects. Specific HDAC3is have been challenging to develop and remain in early-stage pre-clinical development for phase I clinical trials (Mondello et al., 2020). HDAC3 inhibition offers the potential to block the BCL6-HDAC3 complex and restore the key pathways in cell cycle and differentiation inhibited by BCL6 in *CREBBP*-mutant lymphomas, including Ag presentation as well as BCR, NF-kB, and interferon signaling (Mondello et al., 2020). A selective HDAC3i developed by the Broad Institute (BRD3308/OKI422) has demonstrated promising activity both *in vitro* and *in vivo.* HDAC3 inhibition increased H3K27Ac, transcription of B-cell-terminal-differentiation genes, MHC-II expression, and inhibited cell proliferation of lymphoma cell lines even in the absence of a *CREBBP* mutation, although these effects were more marked in *CREBBP*-mutant lines (Mondello et al., 2020). Importantly, HDAC3-specific inhibition induces greater restoration of MHC-II expression compared to pan-HDACis, with particularly robust effects in *CREBBP*-mutant cell lines, and reduced toxicity against T cells (Mondello et al., 2020). In PDX models, HDAC3 inhibition with BRD3308 reduced tumor growth, and induced upregulation of BCL6 target genes and MHC-II expression in the setting of both *CREBBP-*mutant and *CREBBP-*WT diseases (Mondello et al., 2020). Notably, treatment with BRD3308 appeared to improve T-cell mediated tumor recognition and killing, when TILs were co-cultured with DLBCL cells pretreated with BRD3308 vs. vehicle (Mondello et al., 2020). These preclinical experiments suggest that HDAC3-specific inhibition may be an effective therapy for GCB lymphomas, particularly those with *CREBBP-*mutations, by reducing the dominance of BCL6 on transcription programs and improving both terminal differentiation and immunogenicity of tumor cells. In particular, the effects of HDAC3 inhibition in MHC-II expression and T-cell activation speak to its potential as a partner for immunotherapies, such as CAR T cells, ICB, or bispecific antibodies.

### DNA Methyltransferase Inhibitors

Another class of epigenetic drugs—DNA methyltransferase inhibitors (DNMTis)—has shown promising activity against B-cell lymphomas, reaching advanced clinical development. Demethylation at gene promoters induced by DNMTis can result in several beneficial anti-tumor effects: 1) re-expression of tumor suppressor genes, 2) reversal of chemotherapy resistance due to de-repression of SMAD1 and 3) upregulation of the Ag presentation machinery and IFN response genes, which can increase immune sensitivity, thus offering potential for combination with immunotherapy (Almstedt et al., 2010; Chiappinelli et al., 2015). In addition, demethylating agents may have a direct effect on T cells, limiting exhaustion during chronic antigen stimulation. Decitabine was found to prevent the development of exhaustion-associated epigenetic changes in T cells, and this synergized with T-cell reinvigoration upon PD-1 blockade in viral chronic infection mouse models, highlighting the rationale to combine ICB with DNMTis for the treatment of cancer (Ghoneim et al., 2017). Azacytidine and decitabine DNMTis are being tested in several trials in combination with other agents for B-NHLs (e.g. NCT03450343; NCT03579082; NCT01799083). Treatment of high-risk DLBCL patients with azacytidine resulted in reversal of SMAD1 hypermethylation and induction of its expression, which in turn enhanced the response to chemotherapy (Clozel et al., 2013). Accordingly, early promising results were obtained with sequential treatment of azacytidine followed by R-CHOP in high-risk B-cell lymphomas, with several clinical trials in progress including a phase II/III study (NCT04799275) (Clozel et al., 2013; Martin et al., 2021). The impact of these agents in anti-lymphoma immunity remains to be investigated.

### Agents Targeting Lymphoma Metabolism

Understanding metabolic derangements in lymphomas reveals distinct therapeutic vulnerabilities, and one pathway that has shown promise in GCB lymphomas is PI3K, which regulates the PI3K, AKT, and mTOR pathway. Currently, four PI3K inhibitors (PI3Kis) are approved for use in FL and are undergoing study in DLBCL. Idelalisib was the first PI3Ki approved for FL and functions by specifically inhibiting the PI3Kδ isoform and is an oral drug. Following promising results in several phase-I studies, a phase-II trial enrolled patients with indolent lymphomas either refractory to rituximab or alkylating agents or relapsed within 6 months of these therapies (Gopal et al., 2014). While the study did not stratify results by disease subtype, 72 out of 125 patients on study had FL. ORR was 57% (6% CR), with a median PFS of 11 months in this very polyrefractory population (Gopal et al., 2014). The most common grade 3 or higher adverse events were neutropenia, transaminitis, and diarrhea, which may limit the use of this drug in certain cases. Based on this trial, idelalisib was approved for use R/R FL. Idelalisib has also been studied in DLBCL in combination with the Syk inhibitor entospletinib, but the combination was limited by toxicity due to pneumonitis (Barr et al., 2016). Copanlisib is a PI3Ki with activity against PI3Kα and PI3Kδ and is administered intravenously. Following promising phase-I-study results, a large phase-II trial administered copanlisib to patients with R/R indolent or aggressive lymphomas, including 16 FLs and 15 DLBCLs (Dreyling et al., 2017a). ORR in the indolent vs. aggressive subgroup was 44 and 27%, with a median PFS of 270 and 70 days, respectively. A subsequent large phase-II study of copanlisib in R/R indolent lymphomas (FL, n = 104) confirmed the promising results in this population, with 59% ORR (14% CR), 11.2 months median PFS and 22.6 months median DOR (Dreyling et al., 2017b). The most common grade 3–4 adverse events included hypertension, hyperglycemia, leukopenia, and neutropenia, wih nausea, fatigue, and diarrhea common as well. Based on these results, in 2017 copanlisib was approved in R/R FL who have received at least 2 prior therapies. Results of copanlisib monotherapy in DLBCL continued to be disappointing, with a subsequent phase-II study showing an ORR of only 19% (8% CR), although 32% of patients with ABC-DLBCL had a response (Lenz et al., 2020). Most recently, copanlisib has been combined with rituximab in a phase-III trial in patients with R/R indolent lymphomas, including FL, demonstrating 81% ORR (34% CR) in all indolent histologies vs. 48% ORR (15% CR) in the rituximab monotherapy arm. In the 184 FL patients included in the copanlisib-rituximab arm, median PFS was 22 vs. 18.7 months for the 91 FL patients in the rituximab arm (Matasar et al., 2021). However, nearly half of all patients had severe adverse events, including hyperglycemia and hypertension, while less than 20% patients in the single-agent rituximab arm had serious adverse events. Duvelisib is an oral agent that inhibits PI3Kδ and PI3Kγ that has been studied in a phase-II setting in R/R FL with at least 2 prior therapies. 83 patients with FL received duvelisib in a larger study including other indolent NHLs and achieved 42% ORR, including a CR (Flinn et al., 2019). Median PFS in the study as a whole was 9.5 months with a median OS of 29 months. The most common adverse events included diarrhea as well as neutropenia, anemia, and thrombocytopenia. The most recent PI3Ki to be approved is umbralisib, which is an oral agent that inhibits PI3Kδ as well as casein kinase-1 epsilon. In a large phase-II trial, patients with heavily pretreated indolent NHLs were treated with umbralisib monotherapy. In FL patients (n = 117), ORR was 43% with 3% CR rate, a median PFS of 10.6 months and median DOR of 11.1 months (Fowler et al., 2021). The most common toxicities were diarrhea, infection, nausea, neutropenia, transaminitis, and rash. Based on the results of these studies, umbralisib was approved for R/R FL following at least 3 prior lines of therapy. Notably, preclinical studies with a dual PI3K and HDAC inhibitor have revealed the potential of this combination strategy in DLBCLs irrespective or the COO (Mondello et al., 2017) and also in B-cell lymphoma refractory to Bruton’s tyrosine kinase inhibition (Guo et al., 2019).

Another metabolic pathway that may offer therapeutic vulnerability in B-cell lymphomas is the transport of metabolic products/substrates via the monocarboxylate transporter (MCT) family (MCT1, MCT2, MCT3, and MCT4). MCT is a family of transmemmbrane proteins that mediate the bi-directional transport of lactate, pyruvate, short-chain fatty acids and ketones (Halestrap, 2013). MCT1 is a monocarboxylate transporter associated with poor clinical outcomes in DLBCLs (Afonso et al., 2019). MCT1 is activated by the MEK signaling pathway, and both MEK inhbitors (MEKis) and direct MCT1 inhibitors (MCT1is) have been tested in phase-I trials in DLBCL. Selumetinib is a MEKi that was studied in patients with R/R DLBCL, but no patients had objective responses and the drug was poorly tolerated with most patients requiring dose de-escalations (Galanina et al., 2018). Early results of a phase-I study of the MCT1i AZD3965 in R/R DLBCL were recently presented at the ASCO annual meeting, showing CR in one out of 11 patients, with no other clinical responses noted (Halford et al., 2021). Based on these results the MCTi appears to work poorly as a monotherapy, although combinations with other drugs are being considered. Recent preclinical studies in mice have shown that in addition to rewiring the global metabolic activity of cancer cells, MCT1 inhibition can also impact on the TME including angiogenesis, metabolic symbiosis between cancer and stromal cells, and immune suppression (Romero-Garcia et al., 2016). For example, by using non-invasive proton nuclear magnetic resonance (^1^H NMR) spectroscopy (MRS), AZD3965 was found to inhibit tumor choline metabolism *in vivo* with the consequent increases in tumor-infiltrating NK cells and DCs in xenografted lymphoma models, where, however, only innate immune cells could be evaluated. In this study, AZD3965 treatment also showed to upregulate the immune checkpoint PD-L1 on NK cells, providing preliminary evidence for studying the impact of AZD3965 on anti-lymphoma immune responses and in combination with immune-modulating agents (Beloueche-Babari et al., 2020).

As an additional modality to counteract tumor metabolism, the anti-diabetic drug metformin—known to regulate blood glucose by different mechanisms—has started to be explored for B-NHL treatment. Retrospective analyses showed improved survival in diabetic DLBCL patients under metformin treatment during first-line chemotherapy. Moreover, metformin potentiated the anti-tumor activity of rituximab and chemotherapy in lymphoma models, suggesting potential therapeutic effects of metformin against these diseases (Singh et al., 2020).

## T-Cell Targeting Immunotherapies in B-Cell Lymphomas

The development of immunotherapies for B-cell lymphomas including ICB, bispecific antibodies, and CAR T-cell therapies are an incredibly exciting area of innovation and studies in this space have exploded in the past few years. While the full description of this space would deserve a review article unto itself, here we highlight the most important immunotherapy trials in GCB lymphomas, before discussing the potential role of combining epigenetic and metabolic therapies with immunotherapy in these diseases.

### Immune Checkpoint Blockade

In the past decade, ICB targeting PD-1, PD-L1, or CTLA-4 has proven effective against a variety of solid tumor malignancies as well as Hodgkin lymphoma, and there was optimism that ICB would be successful in GCB lymphomas as well. However, trials of ICB in both DLBCL and FL have proven disappointing thus far, underscoring the importance to understand the molecular determinants of this intrinsic immune resistance in these diseases for the design of more effective combination strategies.

Despite the promising results of the anti-PD-1 nivolumab in an initial phase-I study with R/R FL or DLBCL patients with ∼40% ORR and a very manageable toxicity profile (Lesokhin et al., 2016), subsequent phase II-trials showed markedly lower response rates, with 4 and 10% ORR and 2.2 and 1.9 months median PFS in R/R FL and DLBCL, respectively (Ansell et al., 2019; Armand et al., 2021b). Results were similarly poor in a trial with another PD-1 inhibitor, pembrolizumab, in R/R FL (ORR, 11% and median PFS, 3.4 months) (Ding et al., 2017). In R/R DLBCL patients, pembrolizumab was administered following autologous stem cell transplant, but did not meet its primary endpoint of PFS improvement relative to transplant alone (Frigault et al., 2020). The Keynote-013 study (NCT01953692), which has cohorts of pembrolizumab monotherapy in R/R DLBCL and FL, has not yet reported results. Multiagent therapy with pembrolizumab has been shown to increase its efficacy in this setting, with one study combining pembrolizumab and rituximab in R/R FL patients achieving 80% ORR, including 60% CR (Nastoupil et al., 2017).

Atezoliuzumab, a monoclonal antibody targeting PD-L1, has shown some clinical efficacy in combination with the anti-CD20 obinutuzumab in patients with R/R FL or DLBCL, with preliminary data from a phase-I study showing partial responses in one patient with FL and one with DLBCL out of 5 total evaluable patients (Till et al., 2015). Further studies have added lenalidomide to this regimen in FL and showed 85% ORR with a 72% CR rate at most recent update (Salles et al., 2018; Morschhauser et al., 2019). In the front line setting, atezolizumab combined with obinutuzumab and bendamustine for untreated FL showed 80% ORR with 67% CR by Lugano criteria, although 52% of the patients required treatment interruptions including one death due to atezolizumab-related cardiac arrest (Younes et al., 2017). Atezolizumab has also been combined with R-CHOP in untreated DLBCL, with patients receiving atezolizumab consolidation following induction with atezo-RCHOP, with an ORR of 87.5% (77.5% CR) and a 2-years PFS of 75%, although half of the patients discontinued consolidation prior to completion and half had grade 3–4 adverse events during consolidation (Younes et al., 2019).

Overall, these results indicate that PD-1/PD-L1 inhibitors have limited single-agent efficacy in GCB lymphomas, but there remains significant clinical potential when combined with other therapies, including epigenetic modulators and agents targeting tumor metabolism, as discussed below.

### Chimeric Antigen Receptor (CAR) T Cells

CAR T-cell therapies represent an promising area of innovation in the treatment of B-cell lymphomas, in which patient’s T cells are transduced with a viral vector to form a CAR, comprising a tumor-targeting antibody portion linked to TCR intracellular signal transduction domains, which bypasses MHC restrictions for tumor-cell recognition and killing (Ansell and Lin, 2020). These cells are then re-infused into the patient following lymphodepletion. CD19-targeting CAR T cells have gained significant attention, showing long-term, durable efficacy in patients with poly-refractory lymphomas, which traditionally have a very poor prognosis (Crump et al., 2017), and three CD19-targeting CAR T-cell products are currently approved for DLBCL and FL patients. CAR T-cell therapy has its own set of unique toxicities, with cytokine release syndrome (CRS) and neurotoxicity being very common and deserving specific attention (Ansell and Lin, 2020).

Axicabtagene ciloleucel (axi-cel)—a CD19-targeting product which differs from the other approved CAR T-cell products for its CD28 co-stimulatory intracellular domain—was approved for both DLBCL and FL patients. The initial ZUMA-1 trial in R/R DLBCL following two or more lines of prior therapy reported 82% ORRs (54% CR) and long-term durable responses in a subset of patients with CR. Therapy was complicated by grade 3–4 CRS and neurotoxicity in 13 and 28% of patients, respectively (Locke et al., 2019). Subsequently ZUMA-5, which included 124 patients with R/R FL after 2 prior lines of therapy, demonstrated 94% ORR (80% CR) and grade 3–4 CRS and neurotoxicity in 7 and 19% of the patients, respectively (Jacobson et al., 2020). These results led to the approval of axi-cel in both R/R DLBCL and FL following two prior therapies. Axi-cel recently demonstrated preliminary efficacy in untreated double- or triple-hit DLBCL patients or patients with positive PET-CTs following 2 cycles of a rituximab and anthracycline containing regimen, with 93% ORR (80% CR) and grade 3–4 CRS and neurotoxicity in 20 and 27% of patients, respectively (Neelapu et al., 2020). While these results were certainly promising, long-term data will be needed to determine if axi-cel has a role in untreated DLBCL.

The other two CAR T-cell products are currently approved for DLBCL. Tisagenlecleucel (tisa-cel) is a CD3-4-1BB CAR construct that demonstrated 52% ORR (40% CR), with slightly higher rates of grade 3–4 CRS (22%) and similar rates of neurotoxicity (12%) in patients with R/R DLBCLs following two or more lines of therapy, leading to its approval in this setting (Schuster et al., 2019). Lisocabtagene maraleucel is also a CD3-4-1BB CAR T-cell product but it is given at a fixed CD4:CD8 ratio and showed 75% ORR (55% CR) in patients with R/R DLBCL following 2 or more prior therapies, leading to its approval in 2021 for this subgroup (Abramson et al., 2020).

While CAR T-cell therapy is an exciting area for clinical development for the treatment of B-cell lymphomas, there remain several crucial areas for improvement. One of the challenges of adoptive cell therapy is how to improve response duration. CAR T cells as well as effector T cells demand high metabolic support. The CAR structure impacts on CAR-T-cell-product metabolic profiles. CARs with 4-1BB co-stimulatory domains promote OxPhos of fatty acids, central-memory T-cell phenotypes with increased proliferation potential and persistence. CARs with CD28 co-stimulatory domains are more prone to aerobic glycolysis leading to CAR-T-cell products with effector-memory phenotypes (Kawalekar et al., 2016). Epigenetic modifiers can be employed to maintain the essential proportion of stem-cell memory pool in CAR T cells, which can in turn favor longer persistence *in vivo*. For example, treatment with decitabine during CAR-T-cell production was shown to improve CAR-T-cell functional phenotypes, persistence, tumor-homing ability and anti-tumor activity in B-NHL mouse models (Wang Y. et al., 2021). Lastly, prevention of CRS and neurotoxicity remains a critical problem and the use of CAR T-cell products in earlier lines of therapy also an open question that will require further study.

### Bispecific T-Cell Engager (BiTE) Antibodies

BiTE antibodies offer another method for engaging the immune system with lymphoma by creating molecules with separate antigen binding sites targeting both tumor antigen (in the case of GCB lymphoma, CD19 or CD20) and a TCR-activating surface receptor, such as CD3. The toxicities of bispecific antibodies are different from that of traditional chemoimmunotherapy, with many patients developing CRS reminiscent of CAR T-cell toxicity (Ansell and Lin, 2020). BiTE therapy, unlike CAR T-cell therapy, does not require cells to be harvested from patients and is available directly as an off-the-shelf product.

Blinatumomab is a CD3/CD19 BiTE antibody that has gained FDA approval in acute lymphocytic leukemia but has also been studied in R/R DLBCL and FL patients. A phase-I trial recruiting patients with previously treated B-cell lymphomas showed 55% ORR (36% CR) in DLBCL and 80% ORR (40% CR) in FL patients (Goebeler et al., 2016).

Another BiTE product, mosunetuzumab, targets CD3 and CD20 and includes the Fc portion of the antibody to emulate human antibodies more closely. Mosunetuzumab is currently studied in previously treated B-cell lymphomas, and has demonstrated 33% ORR (21% CR) and 61% ORR (50% CR) in DLBCL and FL patients, respectively (Budde et al., 2018). Importantly, toxicity appeared to be more manageable compared to blinatumomab, with no grade 3–4 CRS. A recent update in R/R FL patients after 2 or more prior therapies reported 68% ORR (50% CR) in this patients population, which suggests that mosunetuzumab may be effective even in patients with poly-refractory disease (Assouline et al., 2020). Preliminary data were also published from patients with untreated DLBCL, who could not receive frontline chemotherapy due to age or comorbidities, indicating 55% ORR (46% CR) in this patient subset. Notably, toxicity was manageable with all CRS events being grade one. In a population with limited options, if chemotherapy cannot be given, mosunetuzumab may thus represent a valuable treatment (Olszewski et al., 2020).

Other bispecific antibodies currently in development include odronextuab—a CD3/CD20 IgG4 bispecific antibody—which has shown efficacy in previously treated B-cell lymphomas even in patients refractory to CAR T-cell therapy (Bannerji et al., 2020), and glofitamab—a CD3/CD20 BiTE with a 2:1 antigen configuration to allow for increased tumor antigen binding—which has been combined with obinutuzumab to reduce toxicity (Hutchings et al., 2020a). Epcoritamab—a CD3/CD20 bispecific antibody that is administered subcutaneously—has garnered significant excitement due to its ease of administration, with toxicity profile similar to other BiTE therapies and efficacy in both previously treated DLBCL (67% ORR, 33% CR; including 100% ORR and 50% CR in patients previously treated with CAR T-cell therapy) and FL (100% ORR, 25% CR) (Hutchings et al., 2020b). While clinical data from different BiTE therapies is emerging, the correct sequencing of therapies in more effective combination strategies and the patient populations that may benefit the most from them remain aspects to clarify and important areas of further study.

## Rationale for Novel Immunotherapy-Based Combinations in B-Cell Lymphomas

The explosion in immunotherapy approaches offer potential exciting opportunities for GCB lymphomas patients and clinicians, but still have significant room for improvement. As described above, ICB thus far has shown poor efficacy as monotherapy, but improved efficacy in combination with other therapies and ideal partner drugs have yet to be determined. While CAR T-cell therapy has given durable responses in patients with high-risk, poly-refractory diseases, these patients are the minority and significant room for improvement in long-term efficacy remains (Locke et al., 2019). Similarly, long-term efficacy is an open question for BiTE therapy as well, and many drug partners are being explored in this space too.

Lymphoma resistance to immunotherapies is a critical hurdle and may be related to the immunosuppressed TME and immune escape mechanisms of these diseases. As described in detail above, MHC-II expression is decreased in many GCB lymphomas in association with a worse prognosis (Rimsza et al., 2004). Microenvironmental factors, such as the presence of Tregs, M2 macrophages, MDSCs or low levels of effector and cytotoxic T cells may further prevent anti-lymphoma immune responses (Good et al., 2019; Yan et al., 2019). These factors may be reversed or attenuated by epigenetic and/or metabolic therapy, allowing for increased efficacy of immunotherapies in GCB lymphomas. Moreover, agents targeting epigenetic modifiers and metabolic pathways may exert on-target/off-tumor effects leading to enhanced cytotoxic/functional profiles of anti-tumor T cells, either activated endogenously (e.g. via ICB or BiTE) or administered as cytotoxic T-cell products (e.g. CAR T cells) (Akbari et al., 2021).

EZH2 inhibition has the potential to improve the efficacy of immunotherapies in GCB lymphomas via several mechanisms. In addition to controlling immune-related genes in lymphoma cells, EZH2 is critical for the maintenance of Treg identity after activation, suggesting that EZH2 inhibition may also be able to reduce the suppressive role of Tregs (DuPage et al., 2015). Notably, CTLA-4 blockade with ipilimumab was found to increase EZH2 expression in T cells, including Tregs, and EZH2 inhibition was shown to improve the therapeutic response to anti–CTLA-4 through modulation of the Treg phenotype (Goswami et al., 2018; Wang et al., 2018). H3K27me3 accumulation also suppresses memory T-cell function and drives terminal T-cell differentiation (Gray et al., 2017), suggesting that EZH2 targeted therapy may be able to prolong T-cell functionality, thus providing the rationale for combination with T-cell targeting immunotherapies. EZH2 inactivation also contributes to the recruitment of CD4 and CD8 T cells into the TME by enhancing local Th1-type chemokine production (CXCL9, CXCL10), and loss of EZH2 has also been shown to increase Th2-type cytokine production (Tumes et al., 2013; Peng et al., 2015).

So far, data for combinations of tazemetostat with immunotherapies have been limited. A phase-I study with atezolizumab and twice daily oral tazemetostat until disease progression in previously treated DLBCL patients showed poor results. ORR was a dismal 16%, with 5% CR, although in 5 patients with *EZH2*-mutant disease, 3 responded and 1 had a CR. Although the lack of efficacy precluded this combination from further study, the responses in patients with *EZH2*-mutant lymphomas suggests that a precision-medicine approach targeting this specific patient population may be effective (Palomba et al., 2019).

HDAC3 and KDM5 inhibitors may similarly offer the potential to affect both lymphoma cells and the TME to improve the efficacy of immunotherapy. However, these combinations have only been tested in preclinical settings thus far. HDAC3is demonstrated the ability to induce transcription of BCL6 target genes and MHC-II expression even more so than pan-HDACis, while also increasing T-cell activation against tumor cells (Mondello et al., 2020). Similarly, KMD-5is restore expression of KMT2D- and CREBBP-regulated genes in *KMT2D*-mutant lymphomas, suggesting that these agents may have similar effects in the immune system (Heward J. A. et al., 2021). To add to the list of epigenetic strategies for combination therapies, co-targeting of EZH2 and HDACs has started to be investigated in preclinical models with promising results against EZH2 mutant lymphomas (Lue et al., 2019).

While these therapies remain in very early stages of development, their potential in combination with immunotherapies for the treatment of GCB lymphomas is obvious. Many important questions about targeting specific patient subpopulations and ideal drug combinations/sequences need to be answered for the design of effective epigenetic-immunotherapy approaches. In addition, these approaches may further benefit from incorporating agents targeting lymphoma metabolism, which are becoming clinically available. Considering the effects of cellular metabolism in the activity of epigenetic modifiers and immune microenvironment, starting to investigate rational combinations of metabolic and epigenetic therapies with immunotherapy is the logical next step.

## Conclusions and Perspectives

GCB lymphomas present significant heterogeneity, both across lymphoma samples and between patients. The degree of heterogeneity and derangement from the epigenetic status and dynamics of normal B-cells correlate with disease severity and patient survival (Abramson et al., 2020), adding more complexity to the genetic basis of B-cell lymphomas, with more than 150 genetic driver mutations identified (Chapuy et al., 2018). 85% of all DLBCL cases demonstrate alterations in at least one gene involved in epigenetic remodeling (García-Ramírez et al., 2017; Zhang et al., 2017), such as the histone methyltransferase *KMT2D* (Kawalekar et al., 2016), *EZH2* (Elpek et al., 2007), and the HATs *CREBBP* and *EP300* (Alam et al., 2020). In response to the genetic and epigenetic changes, GCB lymphomas have different ways to escape immune surveillance mechanisms. We described the most relevant ones: 1) defective immune recognition; 2) aberrant immune co-stimulation and co-inhibition; 3) cellular and 4) soluble mediators of active immunosuppression. Notably, *EZH2* GOF and *CREBBP* LOF mutations correspond to downregulation of the Ag presentation machinery (MHC-I/MHC-II), and treatment with EZH2is can reverse these phenotypes (Morin et al., 2010). To add to the complex genetic-epigenetic-immune nature of these tumors, mechanisms of metabolic adaptation are also highly heterogeneous in GCB lymphomas and have the potential to influence the activity of epigenetic modifiers (Jellusova and Rickert, 2017). In earlier studies, the heterogeneous response of DLBCLs to R-CHOP was linked to glycolysis, with the glycolytic enzyme GAPDH being reported to predict for poor therapeutic outcome. Vice versa, GAPDH^low^ lymphomas were found to use other sources metabolic pathways, such as OxPhos and rely on mTORC1 signaling and glutaminolysis (Chiche et al., 2019).

Comprehensive genomic and transcriptomic analyses have allowed to detect subgroups of lymphomas with different metabolic profiles (Caro et al., 2012). However, studies of GCB lymphoma metabolic features are limited and mostly based on gene signatures analyses (Wang H. et al., 2021). Qualitative and quantitative information about preferential substrates and metabolic pathways in GCB lymphoma is required for a comprehensive and potentially actionable framework of the metabolic dysregulations in these diseases. Recently, metabolomic approached based on liquid and/or gas chromatography-mass spectrometry or nuclear magnetic resonance have been explored to identify possible biomarkers for characterization and early diagnosis of the different lymphoma subtypes (Ducker et al., 2017; Schwarzfischer et al., 2017; Barberini et al., 2019). Further deepening our understanding of lymphoma metabolism will be important, as different metabolic states could predict response to therapy and this information may reveal new targets for specific metabolic inhibitors.

The role of the immune microenvironment in GCB lymphomas and B-cell lymphomas overall is also not completely clear. Most findings are based on correlative analyses in patients, with contrasting results in many cases. A major limitation for these studies has been the paucity of faithful syngeneic pre-clinical models where to systematically investigate these mechanisms and the causal implications of specific microenvironmental factors in lymphomagenesis.

Recent advancements in the genetic/epigenetic classification of B-cell lymphomas have allowed to distinguish the oncogenic mutations driving specific disease subtypes more clearly (Chapuy et al., 2018; Mondello et al., 2020; Swenson et al., 2020; Wright et al., 2020; Reimann et al., 2021). This has tremendously advanced the generation of genetically engineered mouse models reproducing the same genetic mutations causing lymphomas in humans and the human diseases (Pasqualucci et al., 2011b; Béguelin et al., 2013; Béguelin et al., 2016; Béguelin et al., 2017; Béguelin et al., 2020). These now constitute an unprecedented resource that will foster and accelerate the study of lymphoma pathogenesis and treatment at different levels. The opportunity to study *in vivo* in a syngeneic setting rational combinations of lymphoma targeted therapies (e.g. epigenetic and metabolic therapies) with immunotherapies will provide fundamental and timely information for rapid clinical translation, including the identification of possible candidate biomarkers to follow in patients. In addition, we will be able to understand the impact of known lymphoma targeted therapies on the microenvironment, providing further rationale for combination with immunotherapy. This knowledge will likely help clarify some of the discordant or unexpected results obtained with some of these treatments in lymphoma patients and will guide the next chapter of precision immune-oncology treatments for these diseases.
